# Analysis of Circulating microRNA Signatures and Preeclampsia Development

**DOI:** 10.3390/cells10051003

**Published:** 2021-04-24

**Authors:** Margarita L. Martinez-Fierro, Idalia Garza-Veloz

**Affiliations:** Molecular Medicine Laboratory, Unidad Academica de Medicina Humanay Ciencias de la Salud, Universidad Autonoma de Zacatecas, Zacatecas 98160, Mexico; idaliagv@uaz.edu.mx

**Keywords:** preeclampsia, miRNA, pregnancy, biomarkers, placenta, VEGF

## Abstract

microRNAs are important regulators of cell processes and have been proposed as potential preeclampsia biomarkers. We evaluated serum microRNA expression profiling to identify microRNAs involved in preeclampsia development. Serum microRNA expression profiling was evaluated at 12, 16, and 20 weeks of gestation (WG), and at the time of preeclampsia diagnosis. Two groups were evaluated using TaqMan low-density array plates: a control group with 18 normotensive pregnant women and a case group with 16 patients who developed preeclampsia during the follow-up period. Fifty-three circulating microRNAs were differentially expressed between groups (*p* < 0.05). Compared with controls, hsa-miR-628-3p showed the highest relative quantity values (at 12 WG = 7.7 and at 20 WG = 3.45) and the hsa-miRs -151a-3p and -573 remained differentially expressed from 16 to 20 WG (*p* < 0.05). Signaling pathways including cancer-related, axon guidance, Neurotrophin, GnRH, VEGF, and B/T cell receptor, were most commonly altered. Further target gene prediction revealed that nuclear factor of activated T-cells 5 gene was included among the transcriptional targets of preeclampsia-modulated microRNAs. Specific microRNAs including hsa-miRs -628-3p, -151a-3p, and -573 were differentially expressed in serum of pregnant women before they developed preeclampsia compared with controls and their participation in the preeclampsia development should be considered.

## 1. Introduction

The microRNAs (miRNA) are small non-coding RNAs of 17–25 nucleotides in length that regulate the expression of target genes by binding to the 3’ untranslated region of the mRNAs [1,2,3]. One miRNA can regulate the mRNA degradation of many genes and thus they have the ability to regulate many biological processes including fetal and placental development [4,5]. In a normal pregnancy, the miRNA expression profile undergoes dynamic changes depending on the gestational stage [6,7,8,9]. Moreover, it has been demonstrated placental miRNAs may be released into the maternal circulation and because they have increased stability compared with that of mRNA, miRNAs have been considered as potential biomarkers for pregnancy monitoring [10].

Since placental and fetal development and vascular homeostasis are disturbed in preeclampsia (PE; a hypertensive disorder of pregnancy defined as new-onset hypertension and either proteinuria or end-organ dysfunction after 20 weeks of gestation) [11,12], important regulatory events such as epigenetic factors and miRNAs could, in turn, become deregulated in PE [12,13]. In fact, aberrant miRNA expression patterns in both the circulation and in the placenta had been associated with PE in previous reports [8,10,14,15,16,17,18]. However, the results of these studies have shown contrasting results; for example, while an up-regulation of serum hsa-miR-520a and hsa-miR-518b at 12–14 weeks of gestation (WG) was reported in women who later developed severe PE [18], in a second study which quantified miRNA profiles in first-trimester serum samples from pregnant women who subsequently developed early PE, significant miRNA signatures were not identified [13]. With the aim to contribute to these efforts, recently, we evaluated the expression profile of 51 members of chromosome 19 miRNA cluster (C19MC) during the first and early second trimesters of pregnancy in serum from women who later developed PE and from normotensive controls. Our results showed circulating levels of hsa-miRs 512-3p, 518f- 3p, 520c-3p, and 520d-3p, were differentially expressed between groups and the serum levels of hsa-miR-518f-3p at 20 WG were useful for identifying women who developed severe PE [19]. Little is known about the contribution of these molecules to the pathogenesis of PE; however, these results demonstrate differential miRNA signatures during the development of pregnancy-related disease. Considering no consensus has been reached regarding which miRNAs are involved in the origin of the clinical manifestations of PE, the aim of this study was to evaluate the circulating miRNA expression profile of 768 miRNAs during the early pregnancy and at the time of PE diagnosis to identify miRNA biomarkers associated with the development of PE. We report differences in circulating miRNA expression patterns over the course of pregnancy; additionally, these patterns were modulated with increased PE severity.

## 2. Materials and Methods

### 2.1. Patients and Study Design

**Figure 1:**
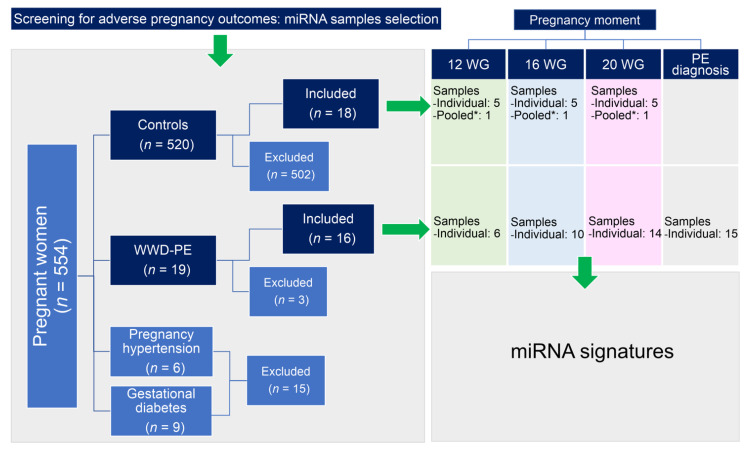
Schematic diagram of the workflow used for the sample selection. A total of 34 participants (16 WWD-PE and 18 controls) donned serum samples, with 24 samples collected at 12 WG, 28 at 16 WG, 32 at 20 WG, and the remaining 15 from the WWD-PE group at the time of PE diagnosis. Each pooled sample (*) was prepared using 20 μL of serum from 13 additional controls with similar characteristics of the WWD-PE. Three WWD-PE were excluded from the study because of the insufficient quantity of samples for the determinations.

This was a retrospective nested cohort case-control study in which the participants were drawn from a cohort of pregnant women who were followed from the first trimester to delivery as part of a screening study for adverse pregnancy outcomes, in Zacatecas Mexico [20,21] (Figure 1).

Sixteen women who later developed PE (WWD-PE) during the follow-up period were selected and matched to 18 women in the cohort who had healthy pregnancies without complications (controls). The PE diagnosis, its severity and onset sub-classifications were established as described in previous investigations [22,23,24,25,26]. Patients with clinical data of other pregnancy-related disorders and/or underlying medical diseases were excluded from the study. In spite of this type of study (retrospective) formal consent is not required, when the recruitment was carried out, all procedures were in accordance with the ethical standards of the institutional research committees and with the Helsinki declaration and its later amendments, and informed consent was obtained from all the participants which agreed with the publication of research data. Participating institutions approved the protocol (protocol ID approvals: ACS/UAZ. Ofc. Nos.0072009, 0062010, HMZ-5020/318/11, HMZ-520/281/11).

### 2.2. Biological Samples

The bank of biological samples of Molecular Medicine Laboratory provided all samples used in the study. Stored miRNA samples (−80 °C) obtained of serum samples from pregnant women were selected. The serum samples were donned at the 12, 16, and/or 20 WG upon enrolling in the study, and at the moment of PE diagnosis (Figure 1). For this study, we used a total of 99 miRNA samples from the 34 participants: 24 from samples donated at 12 WG (6 from WWD-PE and 18 from controls), 28 at 16 WG (10 from WWD-PE and 18 from controls), 32 at 20 WG (14 from WWD-PE and 18 from controls), and the remaining 15 from the WWD-PE group at the time of PE diagnosis [19].

### 2.3. Maternal Serum miRNA Profiling

The miRNA profiling was quantified for each of the 45 PE samples, while only 25 serum samples from 5 normotensive pregnant women were individually quantified at 12, 16, and 20 WG. Additionally, one pooled sample from the 13 additional controls was included for each time point (Figure 1). TaqMan human microRNA array set v2.0 (Applied Biosystems, Foster City, CA, USA) was used for the quantification of the expression of 768 miRNAs according to the manufacturer’s instructions. Quantification cycle (Cq) values were calculated using ViiA™ 7 Software, and the automatic baselines and thresholds were homogenized across multiple runs using Expression Suite software v1.0.3 (Applied Biosystems).

### 2.4. Data Analysis

Comparisons of categorical data among groups were performed using the chi-squared test or Fisher’s exact test. Continuous variables were compared between the groups using the Kruskal–Wallis one-way analysis of variance (ANOVA) on ranks coupled to Dunn’s Method as a multiple comparison procedure. Relative quantity (RQ) of circulating miRNAs was done using the global normalization method, the control group as reference, and a confidence level of 95%. At the time of PE diagnosis, the comparison between the mild and severe PE groups was carried out using the mild PE data as a reference. ΔCq values and standard errors were calculated using Expression Suite software v1.0.3 (Applied Biosystems). The signaling pathways associated with PE development were determined for each pregnancy time point by bioinformatics modeling, using the differentially expressed miRNAs as the input. For this analysis, annotations of the miRNAs of interest had previously been updated using miRBase Tracker (miRNA history). Pathway’s analysis, including miRNA target gene prediction, was performed online via multiple miRNA analysis using DIANA miRPath v2.0 software coupled to the KEGG PATHWAY database (http://www.genome.jp/kegg/pathway.html, accessed on 20 November 2019). In this model, a constant *p*-value threshold of 0.05 and a MicroT threshold of 0.8 were considered.

## 3. Results

### 3.1. General Characteristics of the Study Participants

Thirty-four patients were selected for the study: 16 WWD-PE during the follow-up (cases) and the control group included 18 women with healthy pregnancies without complications. The mean maternal age was 23.5 years old for the WWD-PE group and 23.4 years old for the control group (*p* = 0.913). No differences in clinical data were observed between the study groups (Table 1 and Supplementary Table S1). At the time of the PE diagnosis, the patients showed a mean systolic blood pressure of 151.3 mm/Hg and a mean diastolic blood pressure of 100 mm/Hg, and their urine protein values ranged between 300 and 619.3 mg/dl. 75% of patients had late-onset PE (range: 34–37 WG) and 69% were sub-classified as mild PE.

### 3.2. Early Circulating miRNA Expression Profiles and PE Development

**Figure 2:**
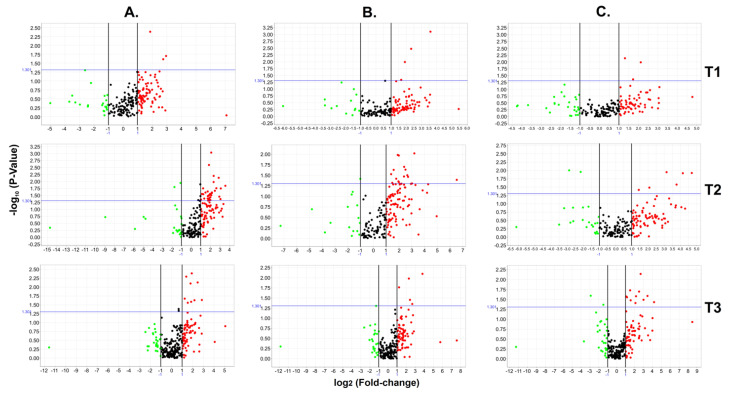
Circulating miRNA expression profiles from the WWD-PE and control groups. The figure shows volcano plots (*p*-value vs. fold change) generated from 768 miRNAs evaluated during the three pregnancy time points (T1–T3: T1 = 12 WG; T2 = 16 WG; T3 = 20 WG) between the WWD-PE and control groups (**A**), the WWD-mild PE and control groups (**B**), and the WWD-severe PE and control groups (**C**). Throughout the analysis, the boundary value for the fold change was 2, and the *p*-value boundary was 0.05. The green and red points correspond to the markers that were under- or overexpressed, while the black points represent miRNAs with expression values outside of the fixed boundaries in the analysis.

Considering a 2-fold change and a *p*-value < 0.05, a total of 53 circulating miRNAs showed differences between the WWD-PE and control groups: 3 at 12 WG, 39 at 16 WG, and 11 at 20 WG (Figure 2 and Supplementary Table S2).

**Figure 3:**
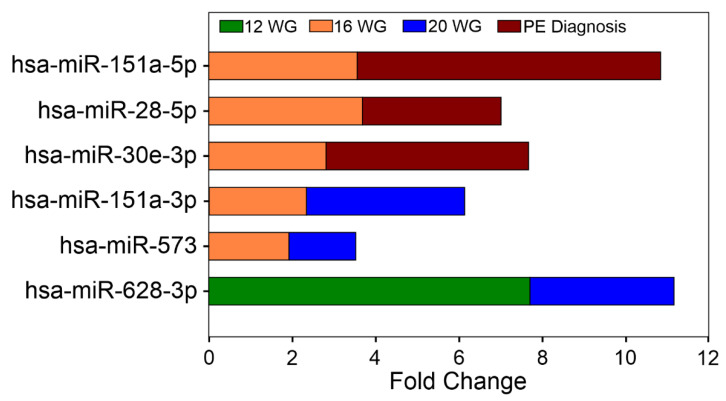
Differentially expressed miRNAs between the groups in at least two of the pregnancy time points evaluated.

The highest RQ values observed for a miRNA at each time point were 7.7 for hsa-miR-628-3p at 12 WG (*p* = 0.02), 11.74 for hsa-miR-142-3p at 16 WG (*p* = 0.014), and 7.22 for hsa-miR-512-3p at 20 WG (*p* = 0.023), respectively. A total of six circulating miRNAs showed differential expression in at least two of the pregnancy time points evaluated (Figure 3); hsa-miR-628-3p had the highest RQ values (RQ values: at 12 WG = 7.7 and at 20 WG = 3.45).

#### Circulating miRNA Expression Signatures and PE Severity

To identify miRNA expression signatures associated with the severity of PE, the participants were classified as women who developed mild PE (WWD-mild-PE), women who developed severe PE (WWD-severe-PE), and controls. The expression of 50 miRNAs differed between the groups (see Figure 2B–C and Supplementary Table S3). At 12 WG, 6 miRNAs were differentially expressed: three between the WWD-mild-PE and control groups (hsa-miR-628-3p, -769-5p, -425-5p), and the remaining three between the WWD-severe-PE and control groups (hsa-miR-365a-3p, -132-3p,-218-5p). At 16 WG, hsa-miR-411-5p was the most significantly overexpressed miRNA in the WWD-mild-PE group (RQ = 9.22; *p* = 0.0095), whereas hsa-miR-197-3p was the miRNA with more significant differences between the WWD-severe-PE and control groups (RQ = 0.14; *p* = 0.0099). At 20 WG, a total of 19 serum miRNAs were differentially expressed between the groups: 6 between the WWD-Mild-PE and control groups, and the remaining 13 between the WWD-severe-PE and control groups (*p* < 0.05). Using the WWD-mild-PE group as a reference, the expression of 20 circulating miRNAs differed between WWD-mild-PE and WWD-severe-PE (*p* < 0.05) at the time of PE diagnosis, with hsa-miR-532-5p as the most significant (*p* = 0.0001) and hsa-miR-106b-3p as the marker with the highest RQ value (RQ = 26.11). Overexpression of the circulating hsa-miR-30e-3p, hsa-miR-28-5p, and hsa-miR-151a-5p markers, which was significant at 16 WG, was also a characteristic finding at the time of the PE diagnosis (Figure 3).

### 3.3. Signaling Pathways Targeted by miRNA Clusters and the Biological Targets

Differentially-expressed miRNAs between the groups with *p*-values < 0.03 were selected to identify the signaling pathways associated with PE development. At 12 WG, the largest number of genes regulated by two of the three differentially miRNAs expressed was grouped into 2 main KEGG pathways: the axon guidance and the prostate cancer signaling pathways (Supplementary Figure S1A). In the gene intersection analysis, *NLK* was identified as the common gene targeted by the 3 miRNAs. At 16 WG, 39 significant KEGG pathways were regulated by at least one miRNA (Supplementary Figure S1B). The MAPK signaling pathway, transcriptional misregulation in cancer, and the GnRH signaling pathway grouped 7 of the 19 miRNAs involved in the analysis (Table 2).

The cluster of hsa-miRs 126-5p, 151a-3p, 199a-3p, 520c-3p, and 27b-3p defined groupings of KEGG cancer-related pathways that included chronic and acute myeloid leukemia pathways and endometrial, non-small cell lung, prostate, and renal cell carcinoma pathways, as well as the cellular junction pathway. Using a cutoff of seven miRNAs, gene intersection analysis identified *UBE2W*, *TAOK1*, and *JMJD1C* as the target genes. Supplementary Figure S1C shows the signaling pathway results for 20 WG. Six of the 9 miRNAs regulated the neurotrophin, axon guidance, and chronic myeloid leukemia pathways, whereas five miRNAs showed significant interactions with the ErbB signaling, transcriptional misregulation in cancer, endometrial cancer, TGF-β, pancreatic cancer, and acute myeloid leukemia pathways. *REV3L* and *ELK4* were the common genes in miRNA intersection analysis of the largest number of miRNAs (6). At the time of PE diagnosis, the neurotrophin signaling pathway had the largest number of genes (19) targeted by seven of the miRNAs involved in the analysis, followed by the pathways regulating the actin cytoskeleton (36 genes) and axon guidance (30 genes), which were regulated by five miRNAs (Supplementary Figure S1D). The target genes that significantly interacted with 5 of the 14 miRNAs evaluated were *KMT2C*, *NFAT5*, *JMJD1C*, and *NR3C1* (Table 2).

## 4. Discussion

In this study, we carried out serum miRNA expression profiling to identify potential miRNAs involved in the development of PE. The circulating miRNA expression profile was evaluated at 12, 16, 20 WG, and at the time of PE diagnosis. Our study identified a total of 53 circulating miRNAs differentially expressed in WWD-PE at the early stages of pregnancy (first and second trimesters) compared with healthy pregnant women. No studies have performed a dynamic determination of the circulating miRNA expression profiles at different time points in early pregnancy; however, two previous reports transversely evaluated the utility of serum miRNAs as early markers of PE. In the first study, Ura B et al. (2014) analyzed miRNAs at 12–14 WG in sera from pregnant women who later developed severe PE [18]. In the second study, Luque A et al. (2014) performed miRNA quantification in first-trimester serum samples from pregnant women who subsequently developed early PE [13]. The results of these studies and ours are divergent, likely because of the differences in the PE patients involved in the study (women who developed severe PE vs. women who developed early PE vs. women who developed PE without stratification), the pregnancy stage in which the miRNA profile was quantified (12–14 WG vs. the first trimester vs. first and second trimester), the biological sample used for miRNA quantification (plasma vs. serum) and the technology used for miRNA detection (microarrays vs. qRT-PCR tools) among others [13,18]. In agreement with before, in our study, circulating miRNA signatures were highly variable when the patients were sub-classified according to disease severity, demonstrating that molecular divergence exists in relation to the current PE stratification criteria. Although our results are not entirely comparable with the previous reports, PE-related modulation of the serum hsa-miR-126 and hsa-miR-144 markers at 12–14 WG, as observed by Ura B et al. (2014) [18], was also detected in this study at 16 WG. Our results showed the hsa-miR-628-3p was the earliest miRNA and had the highest RQ values both at 12 and at 20 WG, positioning them as stable biomarkers associated with the development of PE. In the same sense, the hsa-miRs -151a-3p and -573 remained differentially expressed from 16 to 20 WG demonstrating good potential for an early prediction for PE development.

Is important to note that in our study, differences between the circulating miRNA expression profiles obtained with and without PE classification at 12, 16 and 20 WG were evident. These results highlight the known complexity of PE pathogenesis, yet allowed us to separately identify the individual differentially expressed markers in WWD-severe or mild PE at early pregnancy stages. Although future studies should be performed to validate these markers, our results provide a molecular basis for establishing clinical approaches to improve the prognosis of PE patients. At the time of PE diagnosis, a total of 20 circulating miRNAs were differentially expressed between women with severe and mild PE in this study. The overexpression of hsa-miRs 30e-3p, -28-5p, and -151a-5p was constant, indicating these miRs as both early markers of PE (16 WG) and diagnostic markers of severe PE. Together, these results also provide a new set of markers that may be potentially useful to classify the disease by its severity and it demonstrates that specific miRNAs may have more than one expression peak throughout the pathological stages of the disease.

Abnormal trophoblast implantation is tightly related to the proliferative and invasive capacities of trophoblastic cells [20]. Considering the molecular mechanisms involved during implantation, recent evidence suggests common links between PE and cancer progression pathways [20,27,28,29]. In agreement with these reports and with the findings regarding circulating PE-modulated members of the C19MC cluster (some of which are cancer-related) [30], the signaling pathways modeling results showed that in addition to cancer-related KEGG pathways, Wnt, MAPK, PI3K-Akt, ErbB, GnRH, VEGF, B/T cell receptors, and ubiquitin-mediated proteolysis signaling pathways, were the most commonly modulated by the miRNAs altered in WWD-PE in our study. Aberrant regulation of these signaling pathways has been directly or indirectly associated with PE and/or several types of tumors [29,31,32,33,34,35], emphasizing the involvement and modulation of similar signaling pathways in both pathology types. Further target prediction analysis allowed us to identify a total of 9 miRNA target genes. Circulating miRNAs may be released into the maternal circulation from blood cells or from other tissues that are secondarily affected by the disease, not just by the organ directly associated with the pathology [36,37]. Consequently, both the circulating miRNAs reported in this study and their target genes reflect in a global manner which molecules could be deregulated. Additional studies are necessary to identify the cells or tissues that are responsible for the aberrant miRNA production and to assess whether the release of these miRNA into the maternal circulation results in systemic deregulation of the predicted target genes.

In summary, this study provided the signatures of 768 circulating miRNAs at 4 pregnancy time points, assessing their association with the development and severity of PE, and proposing signaling pathways and transcriptional targets involved in PE based on these altered miRNA signatures. This knowledge provides a new basis for understanding the complex etiopathogenesis of PE, for developing early screening and diagnostic tests, and/or for establishing potential pharmacological therapies for PE.

*Study limitations.* The number of samples included in the study and from which the miRNA signatures were obtained was small and therefore further studies with a larger number of participants are required to validate the miRNAs identified. Clinical data, like gestational age at the moment of delivery, birth weight, or baby sex, were not available and this fact did not allow more robust analysis of its clinical implications and/or to evaluate the influence that these factors had on the variability found in the miRNA signatures in the early stages of pregnancy.

## 5. Conclusions

Serum levels of specific miRNAs implicated in the regulation of cell differentiation, cell proliferation, and/or cancer-related signaling pathways were differentially expressed between WWD-PE and controls. These miRNA patterns were different to those observed according to PE severity and showed usefulness for the identification of altered target genes in PE. Hsa-miR-628-3p was the earliest miRNA with the highest RQ values at 12 WG, demonstrating good potential for an early prediction for PE development. Hsa-miRs -151a-3p and -573 remained differentially expressed from 16 to 20 WG, positioning themselves as stable biomarkers associated with the development of PE. This study provides a new basis for understanding PE etiopathogenesis, for developing early screening tests and/or for establishing potential pharmacological therapies for PE.

## Figures and Tables

**Table 1:** General characteristics of the study population.

Characteristic	WWD-PE (*n* = 16)	Control (*n* = 18)	*p*-Value
Maternal Age (years)	23.5 ± 5.1	23.4 ± 5.8	0.913
Body mass index	28.1 ± 5.5	27.2 ± 4.9	0.639
Number of pregnancies	2 (1–5)	1 (1–4)	0.387
First pregnancy	6 (37.5)	9 (50.0)	0.699
Weeks of gestation at PE diagnosis	34.6 + 5.4	-	-
Early preeclampsia *n* (%)	4 (25.0)	-	-
Severe preeclampsia *n* (%)	5 (31.25)	-	-

**Table 2:** Signaling pathway analysis and miRNA target predictions.

Gestational Age	miRNAs in the Analysis	KEGG Pathway	Enrichment *p*-Value	No. of Target Genes	No. of miRNAs in the Gene Intersection	Gene Name
12 WG	3	Adherens junction	0.0007	1	3	*NLK*
		Wnt signaling pathway	0.0009	1	3	
		MAPK signaling pathway	0.0013	1	3	
16 WG	19	Ubiquitin mediated proteolysis	0.0011	1	7	*UBE2W* *TAOK1* *JMJD1C*
		MAPK signaling pathway	0.0013	1	7	
		Transcriptional misregulation in cancer	0.0013	1	7	
20 WG	9	Fanconi anemia pathway	0.0009	1	6	*REV3L* *ELK4*
		MAPK signaling pathway	0.0009	1	6	
		Metabolic pathways	0.0055	1	6	
		Transcriptional misregulation in cancer	0.0068	1	6	
		HTLV-I infection	0.0079	1	6	
PE diagnosis time ^1^	14	Lysine degradation	1.62 × 10^−5^	1	5	*KMT2C* *NFAT5* *JMJD1C* *NR3C1*
		VEGF signaling pathway	0.0072	1	5	
		B cell receptor signaling pathway	0.0072	1	5	
		T cell receptor signaling pathway	0.0105	1	5	
		Wnt signaling pathway	0.0139	1	5	
		Axon guidance	0.0139	1	5	
		Natural killer cell-mediated cytotoxicity	0.0139	1	5	
		Hepatitis B	0.0139	1	5	
		Transcriptional misregulation in cancer	0.0191	1	5	
		Neuroactive ligand-receptor interaction	0.0229	1	5	
		HTLV-I infection	0.0229	1	5	

^1^ Included only PE cases classified by severe and mild PE. Bold letters indicate the signaling pathways in which the gene intersection is represented by NFAT5.

## Data Availability

Data are contained within the Supplementary Materials. The data presented in this study are available in Tables S1–S3 and Figure S1.

## Supplementary Materials

The following are available online at https://www.mdpi.com/article/10.3390/cells10051003/s1, Table S1: Comparison of the clinical data between the groups at the three follow-up time points. Table S2: Differentially expressed circulating miRNAs between the WWD-PE and control groups during the pregnancy time points evaluated. Table S3: Differentially expressed circulating miRNAs between healthy pregnancies and WWD-PE, classified as mild or severe PE. Figure S1: Signaling pathway analysis.
