# Supplementary material for: Analysis of Circulating microRNA Signatures and Preeclampsia Development
Source: Cells. 2021 Apr 24;10(5):1003. doi: 10.3390/cells10051003 (PMC8145322; doi:10.3390/cells10051003)
Supplement: Supplementary file 1 [file cells-10-01003-s001.zip › 5-Supplemental Material.pdf]

cells MDPI

- Supplementary Material -

Analysis of circulating microRNA signatures and preeclampsia development

Margarita L Martinez-Fierro[1*] and Idalia Garza-Veloz[1]

[1] Molecular Medicine Laboratory, Unidad Academica de Medicina Humana y Ciencias de la Salud. Universidad Autonoma de Zacatecas. Zacatecas, Mexico.

[*]To whom the correspondence should be addressed.

Margarita L Martinez-Fierro. DSc.
Unidad Academica de Medicina Humana y Ciencias de la Salud.
Universidad Autonoma de Zacatecas.
Carretera Zacatecas-Guadalajara Km.6. Ejido la Escondida.
CP 98160, Zacatecas, Mex.
Telephone: +52(492) 9256690 Ext/Fax number:4511.
E-mail: margaritamf@uaz.edu.mx

cells MDPI

Table S1. Comparison of the clinical data between the groups at the three follow-up time points

Clinical finding	12 WG		p value	16 WG		p value	20 WG		p value
	WWD-PE (n = 6)	CONTROLS (n = 18)		WWD-PE (n = 10)	CONTROLS (n = 18)		WWD-PE (n = 14)	CONTROLS (n = 18)	
SBP (mmHg)	113.3 ± 10.3	101.4 ± 12.3	0.052	102.2 ± 12.3	103.2 ± 10.9	0.841	102.7 ± 12.2	103.6 ± 11.5	0.786
DBP (mmHg)	63.3 ± 12.1	67.7 ± 16.2	0.561	62.2 ± 14.8	69.1 ± 11.1	0.215	58.7 ± 12.5	67.4 ± 10.4	0.05
Glucose (mg/dl)	78.0 ± 3.7	82.5 ± 9.2	0.732	82.3 ± 6.9	81.1 ± 11.0	0.796	75.9 ± 9.5	81.1 ± 11.0	0.223
Urine proteins (mg/dl)	Negative	Negative	-	Negative	Negative	-	Negative	Negative	-
Eritrocytes (x10^6/µl)	4.6 ± 0.3	4.9 ± 0.6	0.286	4.4 ± 0.5	4.7 ± 0.4	0.139	4.4 ± 0.2	4.5 ± 0.3	0.399
Hemoglobine (g/dl)	13.6 ± 1.0	14.6 ± 1.0	0.071	13.0 ± 1.4	14.2 ± 0.8	0.063	13.1 ± 0.7	13.6 ± 0.6	0.065
Leucocyte count (x10^3/µl)	8.5 ± 1.7	8.7 ± 1.6	0.827	9.2 ± 1.4	9.1 ± 2.1	0.898	8.8 ± 1.7	9.2 ± 1.9	0.603
Total Cholesterol (mg/dl)	162.9 ± 49.7	180.6 ± 41.2	0.407	187.8 ± 57.8	184.6 ± 35	0.869	196.2 ± 52.9	208 ± 33.5	0.395
HDL (mg/dl)	62.4 ± 16.4	53.7 ± 10.4	0.153	67.9 ± 10.8	60.7 ± 10.7	0.142	65.7 ± 16.2	59.8 ± 10.0	0.232
Triglycerides (mg/ml)	102.1 ±14.7	140.0 ± 43.3	0.15	110.1 ± 18.3	139.4 ± 38.6	0.057	130.6 ± 33.5	152.5 ± 28	0.057

Data are shown as the mean ± SD

cells MDPI

Table S2– Differentially expressed circulating miRNAs between the WWD-PE and control groups during the pregnancy time points evaluated.

Gestational Age	Target	RQ	RQ min	RQ max	p-value
**12 WG**	hsa-miR-628-3p	7.70	1.91	31.03	0.0200
	hsa-miR-766-3p	2.80	1.93	4.07	0.0232
	hsa-miR-323a-3p	6.71	3.36	13.37	0.0246
**16 WG**	hsa-miR-199a-3p	4.24	3.13	5.74	0.0009
	hsa-miR-584-5p	3.59	1.56	8.27	0.0025
	hsa-miR-422a	5.06	2.68	9.55	0.0063
	hsa-miR-411-5p	7.27	4.60	11.51	0.0074
	hsa-miR-27b-3p	5.04	1.40	18.07	0.0104
	hsa-miR-133a-3p	0.46	0.23	0.93	0.0113
	hsa-miR-573	1.92	0.79	4.67	0.0126
	hsa-miR-142-3p	11.74	5.79	23.79	0.0142
	hsa-miR-197-3p	0.30	0.11	0.80	0.0158
	hsa-miR-221-3p	6.11	2.09	17.84	0.0174
	hsa-miR-151a-3p	2.35	0.88	6.27	0.0177
	hsa-miR-34a-3p	8.43	4.37	16.24	0.0177
	hsa-miR-892b	5.69	2.75	11.78	0.0213
	hsa-miR-572	3.28	1.10	9.73	0.0228
	hsa-miR-30e-3p	2.79	0.65	11.98	0.0248
	hsa-miR-1247-5p	4.44	3.42	5.75	0.0248
	hsa-miR-126-5p	4.42	0.99	19.86	0.0249
	hsa-miR-374a-5p	4.39	1.82	10.56	0.0262
	hsa-miR-664a-3p	3.90	0.99	15.30	0.0278
	hsa-miR-18b-5p	2.17	1.16	4.05	0.0280
	hsa-miR-520c-3p	2.37	1.21	4.67	0.0286
	hsa-miR-28-5p	3.70	2.07	6.61	0.0304
	hsa-miR-186-5p	4.24	1.28	14.09	0.0310
	hsa-miR-28-3p	3.49	1.31	9.28	0.0319
	hsa-miR-432-3p	1.95	1.29	2.92	0.0333
	hsa-miR-146b-5p	3.32	1.60	6.88	0.0333
	hsa-miR-490-3p	7.57	1.83	31.30	0.0348
	hsa-miR-126-3p	3.98	0.91	17.50	0.0366
	hsa-miR-19b-3p	3.76	1.30	10.82	0.0385
	hsa-miR-26b-3p	3.28	1.24	8.70	0.0408
	hsa-miR-601	2.55	1.00	6.53	0.0409
	hsa-miR-21-5p	8.27	1.85	36.93	0.0427

	hsa-miR-151a-5p	3.56	1.02	12.47	0.0441
	hsa-miR-146a-5p	2.02	0.56	7.25	0.0444
	hsa-miR-361-5p	3.96	1.98	7.94	0.0460
	hsa-miR-144-5p	3.74	0.78	17.89	0.0462
	hsa-miR-590-5p	1.66	0.95	2.87	0.0465
	hsa-miR-16-5p	3.38	1.26	9.08	0.0483
	hsa-miR-10b-3p	1.86	0.48	7.19	0.0493
**20 WG**	hsa-miR-518f-3p	3.72	1.57	8.83	0.0041
	hsa-miR-302c-3p	2.61	1.08	6.30	0.0051
	hsa-miR-330-5p	5.48	2.55	11.77	0.0074
	hsa-miR-628-5p	3.45	1.94	6.13	0.0079
	hsa-miR-378a-3p	2.36	1.64	3.39	0.0212
	hsa-miR-512-3p	7.22	1.67	31.23	0.0231
	hsa-miR-520d-3p	4.45	2.18	9.09	0.0235
	hsa-miR-142-5p	3.59	1.55	8.33	0.0259
	hsa-miR-19b-1-5p	2.87	1.62	5.08	0.0276
	hsa-miR-875-5p	1.58	1.12	2.23	0.0414
	hsa-miR-573	1.59	1.01	2.51	0.0468
**PE-Diagnosis / Severe PE***	hsa-miR-532-5p	0.07	0.01	0.53	0.0001
	hsa-miR-151a-5p	7.27	5.13	10.31	0.0006
	hsa-miR-106b-3p	26.11	15.36	44.41	0.0035
	hsa-miR-151a-3p	3.79	2.55	5.64	0.0046
	hsa-miR-141-3p	0.14	0.01	3.68	0.0061
	hsa-miR-192-3p	9.95	5.45	18.16	0.0101
	hsa-miR-30d-5p	3.19	2.40	4.23	0.0118
	hsa-miR-191-5p	2.75	1.82	4.16	0.0139
	hsa-miR-28-5p	3.29	2.00	5.42	0.0140
	hsa-miR-30a-5p	2.88	2.03	4.09	0.0194
	hsa-miR-30e-3p	4.89	2.97	8.07	0.0218
	hsa-miR-505-5p	2.34	1.40	3.92	0.0225
	hsa-miR-340-3p	11.20	4.07	30.80	0.0272
	hsa-miR-18a-5p	6.37	2.60	15.65	0.0294
	hsa-miR-93-3p	4.12	2.00	8.49	0.0330
	hsa-miR-183-3p	4.76	1.64	13.78	0.0362
	hsa-miR-22-5p	7.67	3.54	16.62	0.0437
	hsa-miR-942-5p	7.57	4.05	14.15	0.0446
	hsa-miR-409-3p	12.18	3.41	43.47	0.0456
	hsa-miR-425-3p	4.96	1.99	12.35	0.0480

*PE diagnosis, data were obtained considering the Mild PE group as reference.

cells

MDPI

Table S3 – Differentially expressed circulating miRNAs between healthy pregnancies and WWD-PE.

Gestational age	Regulation group	Target	RQ	RQ min	RQ max	p-alue
12 WG	Mild	hsa-miR-628-3p	12.01	6.93	20.79	0.0008
	Mild	hsa-miR-769-5p	4.99	4.49	5.55	0.0033
	Severe	hsa-miR-365a-3p	2.47	1.70	3.60	0.0074
	Mild	hsa-miR-425-5p	3.74	1.16	12.09	0.0102
	Severe	hsa-miR-132-3p	4.34	3.75	5.03	0.0104
	Severe	hsa-miR-218-5p	3.31	1.46	7.47	0.0436
16 WG	Mild	hsa-miR-411-5p	9.22	7.69	11.05	0.0095
	Severe	hsa-miR-197-3p	0.14	0.01	1.60	0.0099
	Mild	hsa-miR-1260a	3.74	2.49	5.62	0.0104
	Mild	hsa-miR-199a-3p	3.98	2.52	6.28	0.0107
	Severe	hsa-miR-603	0.22	0.00	16.82	0.0110
	Severe	hsa-miR-152-3p	8.85	3.08	25.46	0.0113
	Severe	hsa-miR-490-3p	26.04	10.09	67.23	0.0119
	Severe	hsa-miR-579-3p	17.74	16.54	19.03	0.0121
	Mild	hsa-miR-584-5p	5.30	3.04	9.25	0.0199
	Mild	hsa-miR-19b-3p	5.71	3.41	9.57	0.0232
	Mild	hsa-miR-195-5p	3.00	1.60	5.62	0.0234
	Severe	hsa-miR-34a-3p	13.55	7.23	25.38	0.0265
	Mild	hsa-miR-18b-5p	2.62	1.63	4.23	0.0296
	Mild	hsa-miR-15b-3p	6.31	3.30	12.07	0.0314
	Severe	hsa-miR-1247-5p	4.23	2.93	6.09	0.0327
	Mild	hsa-miR-146a-5p	5.26	2.83	9.78	0.0337
	Mild	hsa-miR-27b-3p	6.55	2.98	14.41	0.0344
	Mild	hsa-miR-24-3p	3.46	1.86	6.42	0.0380
	Mild	hsa-miR-133a-3p	0.49	0.20	1.21	0.0382
	Severe	hsa-miR-573	2.69	2.14	3.38	0.0382
	Mild	hsa-miR-520d-3p	92.20	35.94	236.55	0.0403
	Mild	hsa-miR-151a-3p	3.90	1.27	11.92	0.0440
	Mild	hsa-miR-16-5p	3.95	1.65	9.51	0.0459
	Mild	hsa-miR-601	2.78	1.63	4.75	0.0476
	Mild	hsa-miR-664a-3p	8.15	3.12	21.33	0.0493
20 WG	Severe	hsa-miR-518f-3p	6.43	4.05	10.22	0.0072
	Mild	hsa-miR-330-5p	13.83	5.65	33.86	0.0080
	Mild	hsa-miR-19b-1-5p	4.90	2.81	8.54	0.0104
	Mild	hsa-miR-573	2.31	1.59	3.37	0.0173
	Severe	hsa-miR-378a-3p	2.84	1.61	5.01	0.0187
	Severe	hsa-miR-302c-3p	5.70	3.10	10.49	0.0201

Severe	hsa-miR-212-3p	0.13	0.01	2.70	0.0256
Severe	hsa-miR-330-5p	10.97	5.61	21.46	0.0259
Severe	hsa-miR-410-3p	4.49	2.83	7.14	0.0263
Severe	hsa-miR-517a-3p	2.12	1.31	3.43	0.0268
Severe	hsa-miR-215-5p	2.23	1.51	3.30	0.0280
Severe	hsa-miR-365a-3p	8.03	3.68	17.53	0.0323
Severe	hsa-miR-628-5p	2.95	1.51	5.79	0.0334
Mild	hsa-miR-518f-3p	5.34	1.96	14.58	0.0351
Severe	hsa-miR-222-3p	18.25	6.49	51.33	0.0372
Severe	hsa-miR-142-5p	4.30	2.00	9.21	0.0391
Severe	hsa-miR-1290	0.35	0.09	1.35	0.0432
Mild	hsa-miR-520d-3p	6.37	1.66	24.49	0.0443
Mild	hsa-miR-603	0.40	0.25	0.66	0.0495

cells MDPI

Supplementary Figure S1 - Signaling pathway analysis. The heat maps display hierarchical clustering of the targeted pathways at 12 WG (A), 16 WG (B), 20 WG (C), and at the time of the PE diagnosis (D), respectively. Binary values (0: not targeted, 1: targeted) were used during the clustering process
