# Supplementary figures and images for: Analysis of Circulating microRNA Signatures and Preeclampsia Development
Source: Cells. 2021 Apr 24;10(5):1003. doi: 10.3390/cells10051003 (PMC8145322; doi:10.3390/cells10051003)

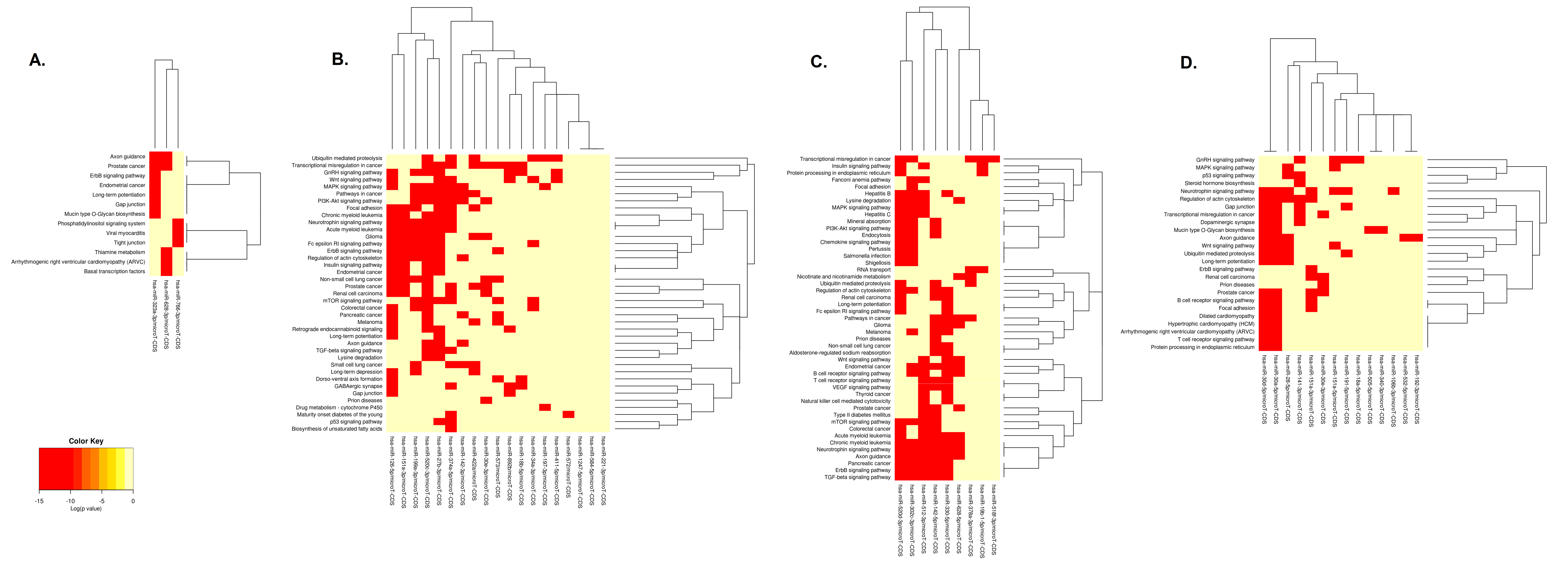

Supplement: Supplementary file 1 [file cells-10-01003-s001.zip › 6-Additional Figure S1-Targeted-Pathways.tif]
